# *UBXN1* is a strong candidate gene in regulation of pork water-holding capacity

**DOI:** 10.5194/aab-64-109-2021

**Published:** 2021-03-23

**Authors:** Jiawen He, Xiangsheng Lin, Haoxin Yang, Ye Tian, Yuelei Zhao, Lifan Zhang, Wei Wei, Jie Chen

**Affiliations:** 1 College of Animal Science and Technology, Nanjing Agricultural University, Nanjing 210095, PR China

## Abstract

The UBX domain containing protein 1-like gene (*UBXN1*) promotes the degradation of myofibrillar proteins during meat maturation, which affects meat water-holding capacity (WHC). This study aims to identify functional mutations in *UBXN1* promoter region, which affects the transcription activity and therefore the WHC. Firstly, we confirmed that the *UBXN1* expression level was positively associated with WHC. Individuals with high and low WHC (n=16 per group) were selected from 168 Duroc × Large White × Yorkshire (D × L × Y) crossbred pigs. The *UBXN1* promoter region was comparatively sequenced using DNA pools from these two groups, and a mutation ca. -379T > G was revealed that had reverse allele distribution. The single nucleotide polymorphism (SNP) was then genotyped in the abovementioned population. TT genotype individuals exhibited higher *UBXN1* mRNA level and higher WHC compared with GG genotype ones. Further luciferase assay confirmed that TT genotype promoter had higher activity. Moreover, the degradation of cytoskeletal framework proteins of muscle cells like *desmin*, *synemin*, *dystrophin*, and *vinculin* was higher in TT genotype individuals than GG ones. In conclusion, we identified a SNP in the *UBXN1* gene promoter that contributes to WHC improvement and pork quality. And *UBXN1* is a strong candidate gene in regulation of pork WHC.

## 1 Introduction

Water-holding capacity (WHC) of meat affects the product quality in terms of meat processing as well as sensory properties of fresh meat cuts. It affects economic outcomes caused by the tissue fluid loss, or drip loss, and subsequently the weight loss. The majority of water in muscle is held within the myofibrils, among the myofibrils, between the myofibrils and the cell membrane (sarcolemma), among muscle cells, and among muscle bundles (groups of muscle cells). The amount and the distribution of water inside the meat have considerable influences on its properties. In the rigor process, the water distribution varies according to the changes produced inside the tissue itself (Honikel and Kim, 1986; Honikel, 2004) and the changes in tissue caused by the degradation of cytoskeletal proteins, which has been suggested as an important process affecting the meat WHC (Huff-Lonergan and Lonergan, 2005). Recent evidence has suggested that degradation of key cytoskeletal proteins by ubiquitin proteasome system and several genes related to ubiquitination were shown to affect muscle and meat properties (Ponsuksili et al., 2008a, b; Damon et al., 2012). Degradation of the myofibrillar proteins reduces the shrinkage of muscle cell and increases WHC (Davis et al., 2004). Desmin is the key member of cytoskeletal proteins. It links myofibrils to each other and cell membrane. Higher level of desmin degradation is correlated with improved WHC during postmortem (Barbut et al., 2008).

The UBX domain is an 80-amino-acid residue module of unknown function present in many eukaryotic proteins. It was originally identified in some proteins implicated in ubiquitination processes (Hofmann and Bucher, 1996). Covalent modification of proteins by ubiquitin is a key regulatory event in a variety of fundamental cellular processes such as targeted protein degradation by the 26S proteasome (Hochstrasser, 1996), Recently, a growing number of small, ubiquitin-like proteins has been demonstrated to be capable of being conjugated to target proteins (Hochstrasser, 2000; Jentsch and Pyrowolakis, 2000). The UBX domain is structurally homologous to ubiquitin and could suggest a role of UBX domain-containing proteins in ubiquitin-related processes including protein degradation, endocytosis, and DNA repair (Buchberger et al., 2001). The *UBXN1* gene encodes a protein that exhibits the ubiquitin regulatory X (UBX domain). It promotes the proteolytic destruction of a subset of proteins in the course of the polyubiquitination process (Buchberger et al., 2001; McNEILL et al., 2004). Interestingly,*UBXN1* is a positional gene for meat quality due to its location on chromosome 2 (SSC2), which contains quantitative trait loci (QTL) for meat, such as drip loss, pH, conductivity and cooking loss (Van Wijk et al., 2006; Heuven et al., 2009). In this study, *UBXN1* was considered a candidate gene for WHC. The promoter region was scanned to reveal mutations that affect *UBXN1* transcriptional activity, and the association between the novel mutations and WHC was verified to develop novel markers for WHC evaluation.

## 2 Materials and methods

### 2.1 Ethics statement

The pig muscle sampling experiment was approved by the Institutional Animal Care and Use Committee of Nanjing Agricultural University (approval number SYXK(SU)2017-0027, Jiangsu, China).

### 2.2 Sample preparation and traits measurement

Animals used for this research were obtained from animals of the 168 Duroc × Large White × Yorkshire (D × L × Y) crossbred pigs, which were sampled from the slaughter house of Haian Country (Haian, Jiangsu Province, China). The longissimus dorsi (LD) muscle was quickly taken from the same place at the last rib. Samples for RNA isolation were stored in liquid nitrogen for later use. At 2 h postmortem, the filter-paper press method (Farouk et al., 2004) was used to measure the volume of losing water from the longissimus dorsi samples held under 35 kg pressure for 5 min. WHC is expressed as the following formula:

$$\mathrm{WHC}\;(\%)=\left(1-\frac{\text{expressible water}}{\text{pre-pressure weight}}\right)\times 100\;\%.$$

### 2.3 DNA pool preparation

**Figure 1:**
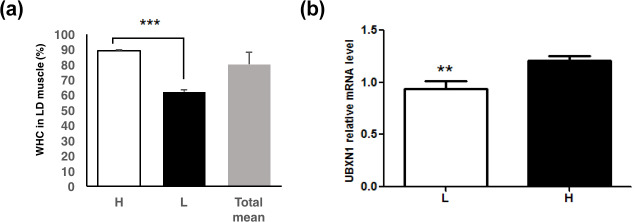
The *UBXN1* expression level in longissimus dorsi muscle of pigs with the low and high WHC. **(a)** The WHC (%) of porcine longissimus dorsi muscle in the low (L), high (H) and total WHC groups. **(b)** The *UBXN1* expression level in the low (L) and high (H) WHC groups. The mRNA levels were normalized to RPLP0 mRNA levels. The data shown in figures are arithmetic means ± standard error of the mean (SEM). n=16 for each group. ${}^{***}$ P<0.001. ${}^{**}$ P<0.01.

Two groups (n=16 per group) with high (89.2 ± 0.4 %) and low (62.1 ± 1.4 %) WHC (P<0.01) were selected from 168 sampled pigs (Fig. 1a). Genomic DNA was extracted from the muscle samples using phenol-chloroform method (Köchl et al., 2005). All absorption ratios (260/280 nm) had to range from 1.8 to 2.0 to meet the requirements. DNA pools of high or low groups were prepared by mixing equal amount of individuals' genomic DNA.

### 2.4 Sequencing and genotyping

In order to identify polymorphisms in the promoter region of *UBXN1* gene, four pairs of primers (Table 1, A–D) were designed to amplify the transcription regulatory region from ca. -2322 to ca. -176 bp (the A of the initiation codon ATG was denoted as +1) for subsequent direct sequencing (Invitrogen, Shanghai, China). The single nucleotide polymorphism (SNP) was genotyped by Sanger sequencing. A 207 bp fragment harboring the SNP ca. -379T > G was amplified with primer E (Table 1, E) in 10 µL reaction mixture which contained 100 ng DNA templates, 5 µL rTaq Premix (Takara, Dalian, China), 0.5 µL of forward and reverse primers (10 µmol) (Invitrogen, Shanghai, China), and 3 µL ddH${}_{2}$O. The cycling protocol was 5 min at 94 ${}^{\circ }$C, 35 cycles of 94 ${}^{\circ }$C for 30 s, 68 ${}^{\circ }$C annealing for 30 s and 72 ${}^{\circ }$C for 30 s, with a final extension at 72 ${}^{\circ }$C for 7 min. Then PCR products were sent to GENEWIZ (Suzhou, China) for Sanger sequencing.

**Table 1:** Primers used in this study.

Primer name	Forward primer $5^{\prime }\to 3^{\prime }$	Application	Length (bp)	Annealing (${}^{\circ }$C)
A	F: 5${}^{\prime }$CATGAGGTTTTGGGTTCGAT3${}^{\prime }$	Promoter clone	875	58
	R: 5${}^{\prime }$GGGTTTGCTTTCTGAGGGA3${}^{\prime }$			
B	F: 5${}^{\prime }$GGGCTAAGCAGTCAACAA3${}^{\prime }$	Promoter clone	691	60
	R: 5${}^{\prime }$CTCCGTGAGAAGTTCCTGT3${}^{\prime }$			
C	F: 5${}^{\prime }$CCAACGGAACGGCATCT3${}^{\prime }$	Promoter clone	539	60
	R: 5${}^{\prime }$AGCCAGTTCCCGATACGAC3${}^{\prime }$			
D	F: 5${}^{\prime }$CAGAGGATGAGCAGGTCAAAGA3${}^{\prime }$	Promoter clone	328	62
	R: 5${}^{\prime }$CGGAAGTGCCCGAATCTGT3${}^{\prime }$			
E	F: 5${}^{\prime }$GGGCGGAGGATGGCTCAACT3${}^{\prime }$	Genotyping	207	68
	R: 5${}^{\prime }$GCCACCGCTCCACATCCTCA3${}^{\prime }$			
F	F: 5${}^{\prime }$GGAGACAAGGGCAGGAGTT3${}^{\prime }$	Real-time PCR	252	58
	R: 5${}^{\prime }$GGGAAGCCACTGAGCAAC3${}^{\prime }$			
G	F: 5${}^{\prime }$TCCAGGCTTTAGGCATCACC3${}^{\prime }$	Real-time PCR	110	62
	R: 5${}^{\prime }$GGCTCCCACTTTGTCTCCAG3${}^{\prime }$			
H	F:5${}^{\prime }$CCCAAGCTTGGGATCGAACCTGCAACC3${}^{\prime }$	Co-transfection	357	64
	R: 5${}^{\prime }$CGGGGTACCACGCCACCGCTCCACAT3${}^{\prime }$			
I	F: 5${}^{\prime }$GCCGAAGAGTGGTACAAGTCAAA3${}^{\prime }$	Real-time PCR	170	62
	R: 5${}^{\prime }$TGCCTCATCAGGGAATCGTTAG3${}^{\prime }$			
J	F: 5${}^{\prime }$GTGGGCGAGGTGCTGGTCTA3${}^{\prime }$	Real-time PCR	156	62
	R: 5${}^{\prime }$GGCTGCGTTCGATGTTCTGG3${}^{\prime }$			
K	F: 5${}^{\prime }$CTGGTGATGGGACAGATAGAG3${}^{\prime }$	Real-time PCR	161	60
	R: 5${}^{\prime }$CATCTCGGAAGAGTGGGTAA3${}^{\prime }$			
L	F: 5${}^{\prime }$GCCAGTTCTCATTTCCGCTAT3${}^{\prime }$	Real-time PCR	158	60
	R: 5${}^{\prime }$CGTCCCAAGAAGTGAGTTGTAAT3${}^{\prime }$			

### 2.5 Real-time quantitative PCR (RT-qPCR)

Total RNA was extracted from LD muscle of different individuals by Trizol reagent (Invitrogen, California, USA). The absorbance of total RNA was measured at 260 nm in an Eppendorf Biophotometer (Eppendorf AG, Hamburg, Germany). All absorption ratios (260/280 nm) must be ranged from 1.8 to 2.0 to meet the requirements. All RNA samples were reverse transcribed in a 10 µL reaction mixture at 37 ${}^{\circ }$C for 15 min and 85 ${}^{\circ }$C for 5 s with 5 × PrimeScript^®^ RT Master Mix (Takara, Dalian, China). The *UBXN1* mRNA relative expression level in LD muscles from GG (n=9) or TT (n=9) individuals was measured by real-time quantitative PCR (qPCR) with primer pair F (Table 1, F). The expressions of *desmin*, *synemin*, *dystrophin*, and *vinculin* in LD muscles from GG (n=13) or TT (n=9) individuals were measured by real-time quantitative PCR (qPCR) with primer pairs I, J, K, and L (Table 1). The PCR amplification was performed in a 20 µL system consisting of 2 µL cDNA (50 ng), 10 µL SYBR Premix Ex TaqTM (Takara), 0.4 µL Rox, and 0.4 µL (10 µM) of each primer. The reactions were performed in an ABI 7900 continuous fluorescence detector (Applied Biosystems), according to standard amplification protocol. RPLP0 expression was measured as an invariant control to normalize the target transcripts using primers named G (Table 1, G). The results of RT-qPCR were analyzed using comparative CT method for mRNA content quantification (Schmittgen and Livak, 2008).

### 2.6 Luciferase assay of promoter activity

The 357 bp fragment which harbors GG and TT genotypes from ca. -677 to ca. -320 was amplified with primer H. The 20 µL amplification system consisted of 200 ng DNA templates, 10 µL rTaq Premix (Takara, Dalian, China), 1 µL of forward and reverse primers (10 µmol) (Invitrogen, Shanghai, China), and 7 µL ddH${}_{2}$O. The cycling protocol was 5 min at 94 ${}^{\circ }$C, 35 cycles of 94 ${}^{\circ }$C for 30 s, 64 ${}^{\circ }$C annealing for 30 s, and 72 ${}^{\circ }$C for 30 s, with a final extension at 72 ${}^{\circ }$C for 7 min. The forward primer was added NheI restriction enzyme cutting site, and reverse primer was added HindIII restriction enzyme cutting site. The two genotype fragments were then cloned into the pGL3-basic vector (Promega, USA) separately. Twenty-four hours before transfection, 293T cells were seeded in each well of 12-well plates. The 293T cells were co-transfected with the constructed reporter plasmid and pRL-TK plasmid (Promega, USA). The transfection system consisted of 1 µg TT or GG genotype reporter plasmid, 0.05 µg pRL-TK plasmid, 100 µL OPTI-MEM, and 3 µL Lipofectamine^®^ 2000 Reagent. Experiments were performed in biological triplicate. Twenty-four hours after transfection, cells were lysed in passive lysis buffer (Promega, USA). Firefly luciferase activity and *Renilla* luciferase activity were measured according to the manufacturer's protocol in three independent experiments (Promega, USA).

### 2.7 Western blotting

Tissue samples were homogenized and lysed by RIPA Lysis Buffer (Beyotime, China). After being bathed in ice for 10 min, the lysates were centrifuged at 4 ${}^{\circ }$C for 20 min at the rotational speed of 12 000 rpm, and the supernate was transferred to a clean tube, and the concentration of the total protein was determined with a BCA Protein Assay Kit (Beyotime, China). Total proteins were denatured under 100 ${}^{\circ }$C for 5 min and separated by sodium dodecyl sulfate-polyacrylamide gel electrophoresis. A prestained protein ladder was used to locate the target bands. Proteins were transferred onto polyvinylidene fluoride (PVDF) membranes (Millipore, USA) by a Mini Trans-Blot Cell (Bio-Rad, USA). The PVDF membranes with proteins were blocked with TBST (20 mM Tris-HCl pH 7.4, 150 mM NaCl, 0.05 % Tween 20) containing 5 % (w/v) bovine serum albumin (BSA), to avoid the nonspecific binding of primary or secondary antibodies. Subsequently they were incubated with primary antibodies at 4 ${}^{\circ }$C overnight. Primary antibodies specific for desmin, vinculin, and GAPDH (Beyotime, China; 1:1000 dilution) were diluted with TBST containing 5 % (w/v) BSA. Membranes were washed 6 times with TBST, and then they were incubated with HRP-conjugated Goat Anti-Rabbit IgG secondary antibody (Sangon Biotech Shanghai, China; 1:2000 dilution in TBST containing 5 % (w/v) BSA) at room temperature for 1 h. After being washed six times with TBST, the signals produced with the BeyoECL Moon chemiluminescence kit (Beyotime, China) were detected under VersaDocTM imaging system (Bio-Rad, USA). The densities of sample bands were analyzed with Quantity One v4.6.2 software (Bio-Rad, USA).

### 2.8 Statistical analysis

The output data of real-time PCR were analyzed by the 2${}^{-\Delta \Delta \mathrm{CT}}$ method (Pfaffl, 2001) for mRNA expression quantification. The evaluation of association between the WHC and *UBXN1* expression level was carried out using the bivariate correlation analysis method. Two average comparisons were performed with one-way ANOVA. Average difference was significant when P<0.05.

The linkage disequilibrium analysis for SNPs ca. -379T > G and ca. -373T > G and deviation from Hardy–Weinberg equilibrium were analyzed with Haploview v4.2 (Broad Institute, USA).

The association between genotypic and phenotypic variation in D × L × Y crossbred pigs was analyzed through using a mixed model (SPSS v20.0, IBM, USA): Y =μ+ G + W + e, where Y is the observation of traits, and μ is the overall mean. Genotype (G) is the fixed effect. Only parts of individuals contain the sex record, so sex is not included in the fixed effect. Slaughter weight (W) is considered co-variable. Random error is denoted by “e”.

The partial promoter sequences containing the mutation site ca. -379T > G were applied to predict potential transcriptional factors using the TFBIND (http://tfbind.hgc.jp, last access: 14 May 2020) online tools.

## 3 Results

### 3.1 *UBXN1* mRNA level is associated with water-holding capacity

Two groups with significantly different WHC (P<0.01) were selected (Fig. 1a). RT-qPCR assay revealed that *UBXN1* mRNA level was higher in the high WHC group (P<0.05) (Fig. 1b). The association analysis revealed that a significant positive correlation existed between *UBXN1* mRNA level and WHC (r=0.44, P<0.05).

### 3.2 A mutation was identified in *UBXN1* promoter

**Figure 2:**
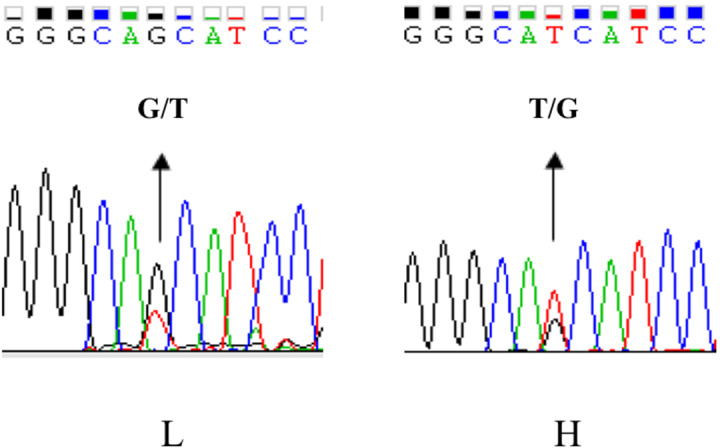
The SNP identification of UBXN1 promoter region by direct sequencing in porcine longissimus dorsi muscle. Partial chromatograms represented the SNP ca. -379T > G in the UBXN1 promoter region.

As *UBXN1* mRNA level was associated with WHC, we try to find out whether a functional mutation exists in the *UBXN1* promoter region, which might lead to expression variations between the high and low groups. Four pairs of primers were used to amplify the promoter region using the high- or low-WHC DNA pools. The fragments were then sequenced directly (Invitrogen, Shanghai, China). Comparative sequencing revealed a novel polymorphism ca. -379T > G in the *UBXN1* promoter region which had opposite allele distribution in the two groups. The ca. -379T was the major allele in the high-WHC group, whereas ca. -379G occurred more frequently in the low-WHC group (Fig. 2).

### 3.3 The ca. -379T > G mutation was associated with water-holding capacity

**Figure 3:**
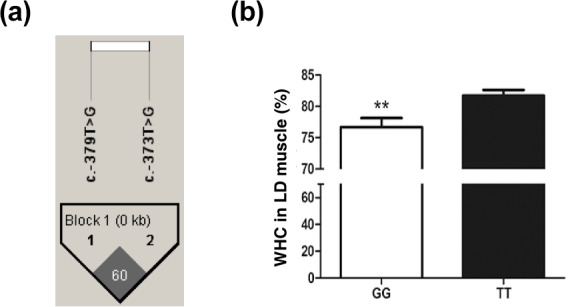
The WHC of longissimus dorsi muscle in pigs with GG and TT genotypes at ca. -373T > G in *UBXN1*. **(a)** Linkage disequilibrium analysis for SNPs ca. -379T > G and ca. -373T > G are based on $r^{2}$ measurement. **(b)** The WHC (%) of porcine longissimus dorsi muscle in GG (n=48) and TT (n=53) genotypes. The data shown in figures are arithmetic means ± standard error of the mean (SEM). ${}^{**}$ P<0.01.

**Figure 4:**
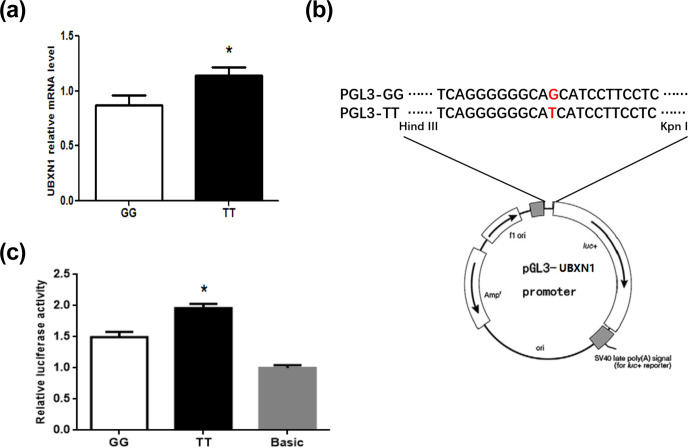
The *UBXN1* expression and transcriptional activity in individuals with GG and TT genotypes. **(a)** *UBXN1* mRNA level of GG (n=9) and TT (n=9) genotypes in porcine longissimus dorsi muscle. ${}^{*}$ P<0.05. **(b)** Schematic illustration of the pGL3-GG/TT-promoter constructs. **(c)** Transcriptional activity analysis of pGL3-GG and pGL3-TT-promoter reporter vectors in 293T cells. The data shown in figures are arithmetic means ± standard error of the mean (SEM). n=3 for each group. ${}^{*}$ P<0.05.

The SNP ca. -379T > G was further genotyped in a population of 168 market pigs (D × L × Y) with the Sanger sequencing method (Fig. 3a). Interestingly, another mutation, SNP ca. -373T > G, was identified close to SNP ca. -379T > G, with an interval of 5 bp. The gene frequency and genotype frequency of the three genotypes (TT, GG, and TG) for two mutations are displayed in Table 2. For both mutations, the genotype distribution was not in Hardy–Weinberg equilibrium. Linkage disequilibrium analysis indicated the incomplete linkage between these two sites (Fig. 4a, $r^{2}=0.605$). Statistical analysis showed significant associations of SNP ca. -379T > G with WHC (P<0.01) and flesh color b (P<0.05) (Table 3). More concretely, WHC of TT genotype was significantly higher than that of GG type (P<0.01, Fig. 3b). This was in accordance with the comparative sequencing result using DNA pools. But associations of SNP ca. -373T > G with all the analyzed traits were not significant (Table 4).

**Table 2:** Different genotypes at ca. -379T > G and ca. -373T > G in *UBXN1* in the population.

	Allele	Gene frequency (%)	Genotype	Number	Genotype frequency (%)
ca. -379T > G	G	0.4851	GG	48	0.2857
			GT	67	0.3988
	T	0.5149	TT	53	0.3155
ca. -373T > G	G	0.3631	GG	30	0.1786
			GT	62	0.3690
	T	0.6369	TT	76	0.4524

**Table 3:** Muscle traits of different genotypes at ca. -379T > G in *UBXN1*.

SNP site	Trait	$N_{1}$	$N_{2}$	Genotype	Mean ± SD
	Leaf fat weight (kg)	143	37	GG	1.13 ± 0.638
			61	GT	1.35 ± 0.835
			45	TT	1.16 ± 0.772
	Water-holding capacity	158	45	GG	0.76 ± 0.100${}^{A}$
			65	GT	0.81 ± 0.068${}^{B}$
			48	TT	0.81 ± 0.068${}^{B}$
	Flesh color L	159	45	GG	42.64 ± 2.769
			66	GT	41.87 ± 2.281
ca. -379T > G			48	TT	42.01 ± 2.840
	Flesh color a	159	45	GG	4.37 ± 1.862
			66	GT	3.81 ± 1.764
			48	TT	3.62 ± 2.004
	Flesh color b	159	45	GG	7.25 ± 1.643${}^{a}$
			66	GT	6.73 ± 1.082${}^{\mathrm{ab}}$
			48	TT	6.59 ± 1.234${}^{b}$
	pH 1	159	45	GG	6.81 ± 0.296
			66	GT	6.72 ± 0.395
			48	TT	6.71 ± 0.364

Multiple hypothesis testing: Bonferroni correction. ${}^{A,B}$ P<0.01. ${}^{a,b}$ P<0.05.

### 3.4 The transcriptional activity of promoter was higher in TT genotype

The qPCR result showed that the expression level of *UBXN1* with TT type was significantly higher than that of GG type (P<0.05) (Fig. 4a). In order to investigate whether this SNP contributes to the transcriptional activity alteration, the reporter plasmid pGL3-GG and pGL3-TT were constructed by inserting a 357 bp promoter fragment harboring different genotypes (Fig. 4b). After co-transfection them with pRL-TK into 293T cells, the dual-luciferase reporter assay showed that TT type had significantly higher activity than GG type (P<0.05) (Fig. 4c).

**Table 4:** Muscle traits of different genotypes at ca. -373T > G in *UBXN1*.

SNP site	Trait	$N_{1}$	$N_{2}$	Genotype	Mean ± SD
	Leaf fat weight (kg)	143	23	GG	1.153 ± 0.719
			55	GT	1.336 ± 0.793
			65	TT	1.181 ± 0.771
	Water-holding capacity	158	28	GG	0.773 ± 0.093
			60	GT	0.799 ± 0.085
			70	TT	0.806 ± 0.072
	Flesh color L	159	28	GG	42.951 ± 2.939
			60	GT	41.755 ± 2.086
ca. -373T > G			71	TT	42.125 ± 2.813
	Flesh color a	159	28	GG	4.046 ± 1.557
			60	GT	4.038 ± 1.859
			71	TT	3.751 ± 2.018
	Flesh color b	159	28	GG	7.203 ± 1.540
			60	GT	6.762 ± 1.230
			71	TT	6.752 ± 1.310
	pH 1	159	28	GG	6.793 ± 0.290
			60	GT	6.751 ± 0.342
			71	TT	6.712 ± 0.400

Multiple hypothesis testing: Bonferroni correction.

### 3.5 Transcription factors prediction

The participation of many transcription factors may have an effect on the process of gene transcription and regulation. The partial promoter sequences containing the mutation site ca. -379T > G were applied to predict potential transcriptional factors. We found that the mutation of this site might have resulted in alternative binding affinities of presumed transcription factors. The presence of the ca. -379G created presumed binding sites for SP1, while NF-κB and YY1 were predicted in the presence of the ca. -379T allele (Table 5).

**Table 5:** Transcription factors of two genotypes on ca. -379T > G in pigs.

Base type	Transcription factor	Score
G	*SP1*	0.806625
T	*NF*-κB	0.808989
	*YY1*	0.790957

### 3.6 The cytoskeletal proteins levels were different within the two genotypes

**Figure 5:**
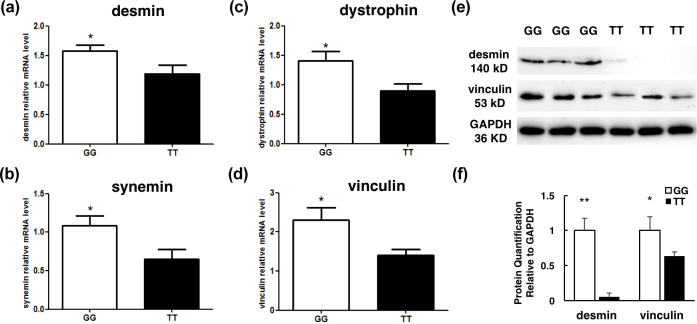
The mRNA expression level detection of cytoskeletal proteins between two groups with different genotypes. **(a–d)** RT-qPCR was performed to detect the mRNA level of *desmin*, *dystrophin*, *synemin*, and *vinculin* in the individuals with the GG (n=13) and TT (n=9) genotypes. **(e–f)** Western blot was performed to detect the protein of desmin and vinculin in the individuals with the GG (n=3) and TT (n=3) genotypes. Results are presented as mean ± SEM ${}^{*}$ P<0.05; ${}^{**}$ P<0.01.

Recent evidence suggests that degradation of key cytoskeletal proteins has an effect on WHC, so we examined the mRNA expression level of cytoskeletal proteins in the individuals with GG genotype and TT genotype. The qPCR showed that the mRNA levels of *desmin*, *synemin*, *dystrophin*, and *vinculin* were significantly lower in the individuals with TT genotype than GG ones (P<0.05, Fig. 5a–d). Moreover, western blot results indicated that the protein of desmin and vinculin in the TT genotype individuals was lower than in GG ones (P<0.05, P<0.01, Fig. 5e–f). That is, the degradation of key cytoskeletal proteins was higher in the individuals with TT genotype than GG ones (P<0.05).

## 4 Discussion

A previous study showed an association of ubiquitination-system-related gene expression with WHC-related traits (Ponsuksili et al., 2008a, b). *UBXN1* is a positional gene for meat quality due to its location on chromosome 2, which contains QTL for meat drip loss, pH, conductivity, and cooking loss (Van Wijk et al., 2006; Heuven et al., 2009). In our study, the expression of *UBXN1* was positively associated with WHC. And a WHC-related SNP ca. -379T > G was identified in the promoter region of the *UBXN1* gene using comparative sequencing. The SNP had been designated as rs327833313 in the NCBI database. Corresponding to the comparative sequencing result, the TT type individuals exhibited a significantly higher expression level of *UBXN1* than GG type ones. This could be explained as that TT promoter possessed higher transcriptional activity than GG promoter, which was further proven by the analysis of luciferase transcriptional activities. Interestingly, study displayed that other polymorphisms of *UBXN1*, ca. 355C > T and ca. 674C > T, were also associated with drip loss; “CT” individuals showed lower transcript abundance and higher WHC compared with “CC” individuals in German Landrace populations, but they showed higher transcript abundance in the commercial crossbreed of Pietrain × (German large white × German Landrace) populations (Loan et al., 2014). Although the transcriptional activities and the effect of ca. -379T > G were divergent with above polymorphisms maybe because of the different genetic background and populations, our results confirm the role of *UBXN1* in regulating pork WHC.

Transcription regulation factors play an important role in regulating gene expression. In some cases, a natural binding site that is created or abolished by a regulatory single nucleotide polymorphism (SNP) within the regulatory regions influences gene expression a lot (Chorley et al., 2008). NFE2 is a member of the Cap'n'Collar (CNC) family of transcription factors, and its protein comprises 373 amino acids (Andrews et al., 1993). The NFE2 protein forms heterodimers with small MAF proteins, and the resulting complex binds to regulatory elements in a large number of target genes. The complex regulatory network that NFE2 participated in includes the regulation of transcription factors such as GATA1 and RUNX1 or the controlling of megakaryocytic and/or erythroid cell function (Gasiorek and Blank, 2015). Based on cell culture studies, it was assumed as a critical regulator of globin gene expression. However, currently there is no evidence to show that this transcription factor has a direct or indirect effect on WHC levels. Further investigations are needed to enlighten the involved molecular mechanisms.

The ubiquitin–proteasome system is one of the major pathways that are responsible for protein turnover or protein degradation in eukaryotes. Interestingly, the ubiquitin–proteasome system also acts during the postmortem period. Until rigor mortis, small amounts of ATP are still present in the muscle cell and activate the ubiquitin–proteasome system that promotes degradation of intermyofibrillar and costameric connections (Sekikawa et al., 2001). Usually, an intracellular protein is ubiquitinated by the covalent attachment of a polyubiquitin chain, and it is destructed into small peptides by the 26S proteasome (Attaix et al., 2002). Degradation of the myofibrillar proteins reduces the shrinkage of muscle cell and increases WHC (Davis et al., 2004). The degraded proteins also provide more space for water. Studies have shown that a higher level of desmin degradation is correlated with improved WHC during postmortem (Barbut et al., 2008). Similar phenomena have been observed in enhanced pork loins where reduced degradation of desmin was associated with increased purge loss (Davis et al., 2004). Furthermore, a current hypothesis proposes that proteolysis of key muscle proteins, such as intermediate filament protein and desmin, may minimize the loss of water from cell interior to the drip channels (Morrison et al., 1998; Melody et al., 2004; Huff-Lonergan and Lonergan, 2005) caused by lateral shrinkage of myofibrils in postmortem muscle (Diesbourg et al., 1988). Myofibril shrinkage corresponds to the constriction of entire muscle cell, and the linkages between adjacent myofibrils as well as between myofibrils and membrane are made up of the cooperation of several proteins including desmin, filamin, synemin, dystrophin, talin, and vinculin (Greaser, 1991). According to our results, the degradation of key cytoskeletal proteins was higher in the individuals with TT genotype than GG ones, corresponding to the expression pattern of *UBXN1*, indicating the role of *UBXN1* in reducing the shrinkage of myofibrils, and causing the increase of WHC by increasing the degradation of cytoskeletal proteins.

In summary, our results identified a ca. -379T > G polymorphism in the promoter region of *UBXN1* gene that was associated with WHC in the D × L × Y crossbred pigs. The two kinds of natural genotypes had allele-specific effects on *UBXN1* promoter activity and mRNA expression. These results provide evidence for the effect of *UBXN1* genetic variation on WHC and offer a promising genetic marker for the improvement of meat quality in pigs. In combination with previous functional polymorphisms, *UBXN1* is considered to be a strong candidate gene in regulation of pork WHC.

## 5 Conclusions

Water-holding capacity is a meat quality trait that affects economic outcomes caused by the tissue fluid loss and subsequently the weight loss. The research of functional single nucleotide polymorphism of *UBXN1* related to ubiquitination can enable one to better understand the mechanisms in degradation of myofibrillar proteins underlying water-holding capacity. The present study indicates that the mutation ca. -379T > G in the *UBXN1* promoter is associated with promoter activity, *UBXN1* mRNA level as well as WHC. Therefore, it potentially contributes to WHC improvement. It helps to find a promising marker for the selection of pork quality and increase economic benefits of the pork industry. And on the basis of available evidence, *UBXN1* is considered to be a strong candidate gene in regulation of pork water-holding capacity.

## Data Availability

No data sets were used in this article.
